# Cisplatin-Induced Non-Oliguric Acute Kidney Injury in a Pediatric Experimental Animal Model in Piglets

**DOI:** 10.1371/journal.pone.0149013

**Published:** 2016-02-12

**Authors:** Maria José Santiago, Sarah Nicole Fernández, Alberto Lázaro, Rafael González, Javier Urbano, Jorge López, Maria José Solana, Blanca Toledo, Jimena del Castillo, Alberto Tejedor, Jesús López-Herce

**Affiliations:** 1 Paediatric Intensive Care Department. Hospital General Universitario Gregorio Marañón, Instituto de Investigación Sanitaria Gregorio Marañón, Hospital General Universitario Gregorio Marañón, Madrid, Spain; 2 Spanish Health Institute Carlos III Maternal, Child Health and Development Network, Madrid, Spain; 3 Complutense University of Madrid, Madrid, Spain; 4 Laboratory of Renal Physiopathology, Department of Nephrology, Instituto de Investigación Sanitaria Gregorio Marañón, Hospital General Universitario Gregorio Marañón, Madrid, Spain; Emory University, UNITED STATES

## Abstract

**Objective:**

To design an experimental pediatric animal model of acute kidney injury induced by cisplatin.

**Methods:**

Prospective comparative observational animal study in two different phases. Acute kidney injury was induced using three different doses of cisplatin (2, 3 and 5 mg/kg). The development of nephrotoxicity was assessed 2 to 4 days after cisplatin administration by estimating biochemical parameters, diuresis and renal morphology. Analytical values and renal morphology were compared between 15 piglets treated with cisplatin 3 mg/kg and 15 control piglets in the second phase of the study.

**Results:**

41 piglets were studied. The dose of 3 mg/kg administered 48 hours before the experience induced a significant increase in serum creatinine and urea without an increase in potassium levels.

Piglets treated with cisplatin 3 mg/kg had significantly higher values of creatinine, urea, phosphate and amylase, less diuresis and lower values of potassium, sodium and bicarbonate than control piglets. Histological findings showed evidence of a dose-dependent increase in renal damage.

**Conclusions:**

a dose of 3 mg/kg of cisplatin induces a significant alteration in renal function 48 hours after its administration, so it can be used as a pediatric animal model of non-oliguric acute kidney injury.

## Introduction

Acute kidney injury (AKI) is a clinical syndrome that can be caused by multiple insults affecting renal circulation or parenchyma [1]. The most common causes of AKI in children are vascular insults secondary to ischemia or hypoxia due to shock, followed by toxics, systemic diseases and intrinsic renal diseases [2,3].

Studying AKI in humans can be complicated, and animal models are required in many occasions in order to assess pathophysiological mechanisms and to evaluate new diagnostic and therapeutic methods [4–12].

Most drug or toxic-induced AKI animal models have used rats for its simplicity and ready availability [4, 10–12]. Nevertheless, rats are very distant to human models. Pigs are more akin to humans, renal blood flow can be measured and the effect of renal replacement therapies can be assessed more easily.

Most studies of AKI in animal models have used ischemia [7,8], rhabdomyolysis [9]or different toxics such as gentamicin [10] or warfarin [11], which induce an oligoanuric AKI.

Cisplatin is used in humans for treating cancer and it is known to produce non-oliguric AKI at high doses.

Cisplatin is freely filtered at the glomeruli and it is taken up by renal tubular cells reaching the proximal tubular inner medullae and outer cortices. Therefore, these areas are the dominant sites for cisplatin-induced renal injury, although other tubular areas such as the distal and collecting tubules are also affected. The mechanisms of cisplatin-induced nephrotoxicity are complex. Several mechanisms including oxidative stress, DNA damage, and inflammatory responses are associated with cisplatin-induced nephrotoxicity [13–15].

There are some animal models of cisplatin induced AKI in rats [16–19] and monkeys [20], but not in pigs.

The aim of our study was to design a pediatric animal model of toxic, non-oliguric AKI in piglets using cisplatin in order to assess the effects of continuous renal replacement therapies on urine output.

## Materials and Methods

A prospective randomized animal study was carried out. The experimental protocol was approved by the experimental protocol was approved by the Gregorio Marañon University Hospital Ethics Committee for Animal Research. Animal studies were conducted in the Experimental Medicine and Surgery Unit of the Gregorio Marañón University Hospital in Madrid, Spain. Animal care was carried out by qualified technicians supervised by veterinarians. International guidelines for ethical conduct in the care and use of experimental animals were applied throughout the study. Animal studies were conducted in the Experimental Medicine and Surgery Unit of the Gregorio Marañón University Hospital in Madrid, Spain. International guidelines for ethical conduct in the care and use of experimental animals were applied throughout the study.

The study was divided in two phases; first, 11 healthy 2-to-3-month-old Maryland pigs with a mean weight of 9.1 ± 1.6 kg were used. The gender of the animals was 54% males, 46% females. Piglets were premedicated with intramuscular ketamine (15 mg/kg) and atropine (0.02 mg/kg). After canalization of a peripheral vein in the ear, cisplatin was administered. Intravenous administration of cisplatin requires the use of gloves and a laboratory coat due to its toxicity, further handling of the animal does not require any special precautions. The first cisplatin dose (5 mg/kg) was chosen according to that used in rat models [18]. Cisplatin doses and intervals are summarized in Table 1. The volume administration of cisplatin solution was between 40 and 60 ml, depending on the weight of the piglet (solution of 0.5 mg/ml) and the dose administered. Two to four days later animals were anesthetized with boluses of propofol (5 mg/kg), fentanyl (5 μg/kg) and atracurium (0.5 mg/kg) for oral endotracheal intubation. Diuresis was measured directly from the bladder through a suprapubic cystostomy and catheterization. It was measured in the second part of the study, starting at 48 hours after cisplatin administration until the end of the experiment (for about 8 hours).

**Table 1:** Cisplatin dose, interval and laboratory data.

Numberof piglets	Dose (mg/kg)	Interval (days)	Creatinine (mg/dl)	Urea (mg/dl)	Potassium (mEq/L)	Phosphate (mg/dl)	Amylase (U/L)
1	5	3	5.5	409	10.9	14.8	5770
1	2	4	1.1	46	4.3	6.8	1198
1	3	4	3.6	174	4.6	20.8	3261
4	3	2	3.3	179	4.5	15.1	4238
2	3	1.5	1.0	82	4.3	13	4230
2	0	-	0.6	33	4.8	9.6	938

Laboratory data shows measurements from after 48 hours of cisplatin administration and before starting CRRT.

Plasma creatinine, urea, potassium, chloride, sodium, calcium, phosphate, amilase, serum cystatin C, N-GAL and diuresis were measured. Blood gases were analyzed using the GEM Premier 3000^®^ blood gas analyzer (Instrumentation Laboratory, Lexington, Kentucky, USA).

Surgical removal of the kidney was performed at the end of each experiment.

Kidney samples were snap-frozen in liquid nitrogen and stored at -80° or fixed in 4% paraformaldehyde (24 h) and paraffin-embedded. For light microscopy renal histopathological studies, paraffin-embedded renal sections (4-μm thick) were stained with haematoxylin-eosin (Sigma^®^ Aldrich, St Louis, MO).

In the second phase, the effectiveness of the dose and interval of cisplatin administration was assessed in 15 piglets, comparing these results with the same number of control piglets that had not received cisplatin in the setting of another study evaluating the effectiveness of continuous renal replacement therapies in a pediatric model.

Piglets were euthanized at the end of the experiment by administering supra-anesthetic doses of fentanyl and propofol immediately followed by rapid intravenous infusion of potassium chloride (4 mEq/kg).

Statistical analysis comparing values between piglets that had and had not received cisplatin was performed using the Wilcoxon test. A p value of <0.05 was considered statistically significant.

## Results

Table 1 shows kidney function values at baseline after different doses of cisplatin.

Doses of 3 mg/kg administered 48 hours earlier induced a significant increase of plasma urea and creatinine without a significant alteration in potassium levels. Lower doses (2 mg/kg) or shorter intervals (1.5 days) did not induce a significant alteration in kidney function, and a higher dose of cisplatin (5 mg/kg) induced severe AKI with increased potassium levels (10.9 mEq/L).

Table 2 compares renal function parameters between the 15 piglets that were treated with 3 mg/kg of cisplatin and the control group. Measurements are from after 48 hours of cisplatin administration and before starting CRRT. Cisplatin piglets had significantly higher levels of creatinine, urea, phosphate, amylase and lipase and lower urine output and lower plasma potassium, sodium and bicarbonate levels than the control group.

**Table 2:** Comparison in renal function between cisplatin and control piglets before starting CRRT.

Group (Number of pigs)	Cisplatin (15)		Control (15)		p
	Median	IQR	Median	IQR	
Creatinine (mg/dl)	3.3	2.7–3.8	0.5	0.5–0.6	<0.001
Urea (mg/dl)	172	138–209	37	33–44	<0.001
Potasium (mEq/L)	4.2	3.7–4.6	5.3	4.8–5.5	0.002
Phosphate (mg/dl)	14.9	14.2–16	11.6	10.7–12.6	<0.001
Sodium (mEq/L)	138	135–139	140	139–142	0.019
Chloride (mEq/L)	92	90–97.5	100	98.5–101	
Calcium (mg/dL)	8.8	8.6–9.4	9.7	9.4–9.8	0.002
Magnesium (mg/dL)	2.4	2.4–2.5	2.5	2.3–2.6	0.781
Bicarbonate (mEq/L)	22	19.9–24	29.2	28.5–31	<0.001
Amylase (mg/dl)	3725	2692–5752	651	595–1341	<0.001
Lipase (mg/dl)	50	27–66	6	5–6	<0.001
ALT (U/L)	30	26–34	28	24–32	0.289
Diuresis (ml/h)	20	6–35	46	30–60	0.005

IQR: interquartile range (p25-p75). ALT: Alanine Aminotransferase. Laboratory data shows measurements from after 48 hours of cisplatin administration and before starting CRRT. Diuresis refers to the urinary output in the first hour, before starting CRRT.

Plasma creatinine levels were above 2 mg/dl in 86.7% and urea levels were above 60 mg/dl in 100% of the piglets treated with 3 mg/kg of cisplatin, whereas none in the control group reached such high levels of creatinine and urea (p<0.001).

Baseline N-GAL serum levels were significantly higher in the cisplatin group than in the control group (427 ng/ml (SD 257) vs 163 ng/ml (SD 40), p 0.001). Nevertheless, there were no statistically significant differences in cystatin C levels (2.61 mg/L (SD 2.5) vs 1.83 mg/L (SD 1), respectively. p 0.16).

Cisplatin piglets also had lower urea, sodium and chloride urinary excretion than controls (Table 3). Potassium urinary excretion was higher in cisplatin piglets with a higher transtubular potassium gradient than control piglets (Cisplatin group: 11.7 versus Control group: 5.0, p <0.001).

**Table 3:** Comparison in urine values between cisplatin and control piglets before starting CRRT.

	Cisplatin		Control		p
	Median	IQR	Median	IQR	
Osm U (mOsm/L)	505.5	453–619	720	625–749	0.003
Sodium U (mmol/L)	19	13–22	67.5	40–95	<0.001
Potasium U (mmol/L)	100	80.2–100	67.4	64.2–72.1	0.006
Cloride U (mmol/L)	20	20–24	65	53–79	<0.001
Calcium (mg/dL)	1.25	0.65–3.75	3.5	1.7–4.1	0.0961
Creatinine U (mg/dL)	58.9	37.9–77.7	77	56–80.2	0.281
Urea U (mg/dL)	1079	853–1247	1912	1690–2343	0.001
	Mean	SD	Mean	SD	p
TTKG	11.7	4.2	5.0	1.7	<0.001

Osm U: Urinary osmolarity. TTKG: Transtubular potassium gradient.

In the period between cisplatin administration and evaluation, the piglets did not show any systemic symptoms (no weight loss, nausea or vomiting). Nor did they show local symptoms (no skin rash or neurological symptoms, liver function and coagulation were normal).

After 6 hours of CRRT, creatinine levels remained stable in the control group (mean baseline: 0.58 mg/dl (SD 0.09) vs mean 6 hours: 0.57 mg/dl (SD 0.13), p 0.67) whereas urea dropped from 39 mg/dl (SD 7) to 35 mg/dl (SD 10), p 0.04. In the cisplatin group however, a significant drop in both creatinine (3.1 mg/dl, (SD 0.26) vs 2.5 mg/dl (SD 0.23), p<0.001) and urea (168 mg/dl (SD 45) vs 133 mg/dl (SD 39), p< 0.001) was observed.

**Fig 1:**
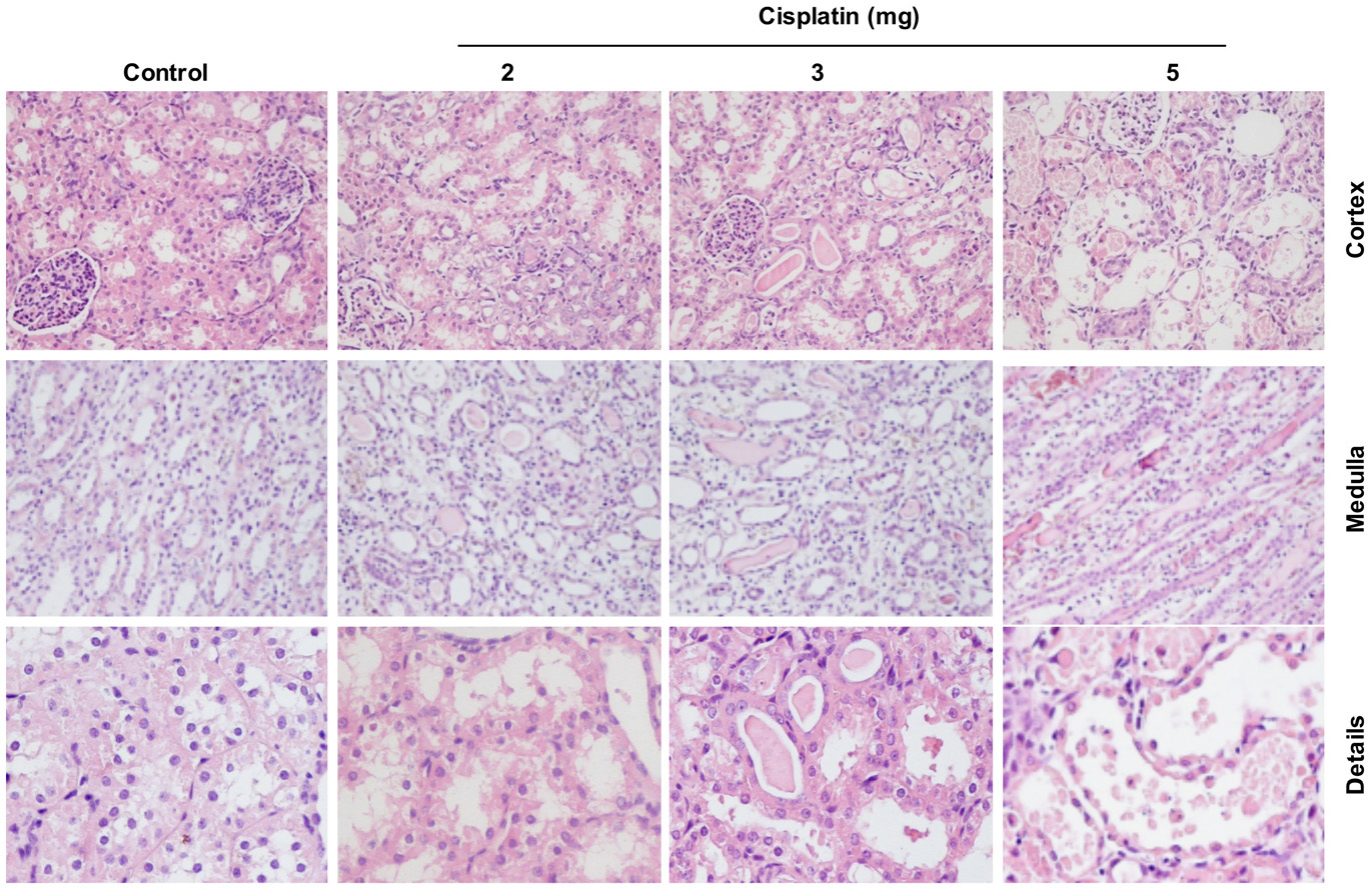
Histopathological study of hematoxylin-eosin-stained pig kidney sections. Representative images of the renal pathology (cortex and medulla—magnification X20- and details—magnification X60-) on day 2 after administration of 2, 3 or 5 mg/kg cisplatin. “Details” refers to cortex details.

The toxic effect of cisplatin was also confirmed by the detection of morphologic abnormalities in kidney slices. The healthy control cortex histology was altered after the administration of 2, 3 and 5 mg/kg cisplatin showing a dose-dependent increase of renal damage. Cisplatin piglets exhibited acute structural damage with tubular necrosis, swelling and tubular dilation, loss of brush border membrane, extensive epithelial vacuolization, hyaline casts (protein aggregates) and cell debris detachment. These effects were dramatically demonstrated with 5 mg/kg cisplatin, whereas these findings were much milder with the 2 mg/kg dose. Piglets with the intermediate dose of 3 mg/kg also showed severe structural damage, similar to that of the 5 mg/kg cisplatin. Moreover, kidney slices of piglets treated with doses of 5 and 3 mg/kg showed marked inflammatory cell infiltration in interstitial areas that are not apparent with 2 mg/kg cisplatin. Structural damage caused by cisplatin takes place mainly in cortical tubule cells, specifically in renal proximal tubular epithelial cells, although the damage is also reflected in the medulla where protein casts were observed (Fig 1).

## Discussion

Most AKI animal models use ischemic or toxic insults to induce AKI, which is oliguric [4–12]. Most of them have been performed in rats, in which the effect of diuretics or renal replacement therapies cannot be tested [16–19]. This is, to our knowledge, the first animal model of pediatric cisplatin induced non-oliguric AKI in piglets. Our study shows that a dose of 3 mg/kg administered 48 hours earlier induces significant renal failure in pediatric piglets with a decrease in urinary output without any significant electrolyte imbalances. Renal function biomarkers other than creatinine and urea, such as N-GAL and cystatin C, were tested in our model. Cisplatin treated pigs presented higher values of both markers, but significant differences were only found in N-GAL at baseline. This is probably due to the small sample size.

Even though urinary output was decreased by 50% in cisplatin piglets, it was still 20 ml/hour, which is about 2 ml/kg/h. Decreased urinary output can be partly explained because, even though piglets were allowed to drink “*ad libitum*”, fluid intake was probably insufficient. There are indeed statistically significant differences in electrolyte values as shown in Table 2, but these differences are not clinically relevant at all (i.e. potassium of 4.2 vs 5.3, sodium 138 vs 140, calcium 8.8 vs 9.7). All these values are within normal limits. Acute pancreatitis is a potential complication of this treatment, which is why amylase and lipase levels were determined. Lipase levels are more sensitive and specific for diagnosing acute pancreatitis, and lipase levels were normal [21] in all except one piglet in the cisplatin group that showed lipase levels of 2.5 times the normal limit. Since none of the piglets had any clinical manifestations of pancreatitis and lipase levels were normal, we assume that AKI was not influenced by acute pancreatitis in any of the piglets. There was no evidence of organ injury other than AKI (liver, coagulation, CNS, etc).

This dose of cisplatin could be used in pediatric animal models of toxic non oliguric AKI to assess the effect of renal replacement therapies or different drugs on renal function and urinary output.

The main advantages of this model are its simplicity, since only one intravenous dose of cisplatin is required, and its reproducibility (as observed in the second part of the study). Even though handling cisplatin for intravenous administration requires the use of gloves and a laboratory coat due to its toxicity, further handling of the animal does not require any special precautions.

Anatomopathological findings show that this dose of cisplatin induces AKI with signs of kidney injury such as cell loss, tubular dilation, protein casts that obstruct tubules and interstitial inflammatory infiltration.

## Limitations

The first part of the study, in which different doses and time intervals of cisplatin were tested, was performed in very few animals due to ethical reasons, since the number of animals must be as reduced as possible. Nevertheless, the following part of the study confirmed that the dose of 3 mg/kg induced AKI in all the piglets that received it.

## Conclusions

A dose of 3 mg/kg of cisplatin produces a significant alteration in renal function without affecting urinary output 48 hours after its administration, and it can be used for non-oliguric pediatric AKI animal models.
