# Correction: Cisplatin-Induced Non-Oliguric Acute Kidney Injury in a Pediatric Experimental Animal Model in Piglets

**DOI:** 10.1371/journal.pone.0207547

**Published:** 2018-11-09

**Authors:** Maria José Santiago, Sarah Nicole Fernández, Alberto Lázaro, Rafael González, Javier Urbano, Jorge López, Maria José Solana, Blanca Toledo, Jimena del Castillo, Alberto Tejedor, Jesús López-Herce

In the Funding section, the first grant number from the funder Carlos III Institute of Health, Spain, and Comunidad Autónoma de Madrid (Consorcio para la Investigación del Fracaso Renal Agudo–CIFRA-) S2010/BMD-2378 is listed incorrectly. The correct grant number is: PI12/01328.
